# Immunohistochemical analysis of the expression of MAGE-A and NY-ESO-1 cancer/testis antigens in diffuse large B-cell testicular lymphoma

**DOI:** 10.1186/1479-5876-11-123

**Published:** 2013-05-16

**Authors:** Tvrtko Hudolin, Zeljko Kastelan, Ivana Ilic, Katarina Levarda-Hudolin, Nikolina Basic-Jukic, Malte Rieken, Giulio C Spagnoli, Antonio Juretic, Chantal Mengus

**Affiliations:** 1 Department of Urology, Zagreb University Hospital Center, Zagreb, Croatia; 2 Department of Pathology, Zagreb University Hospital Center, Zagreb, Croatia; 3 Department of Prosthetic Dentistry, School of dental medicine, Zagreb University Hospital Center, Zagreb, Croatia; 4 Department of Dyalisis, Zagreb University Hospital Center, Zagreb, Croatia; 5 Departments of Surgery and Biomedicine, Basel University Hospital, Basel, Switzerland; 6 Department of Oncology, Zagreb University Hospital Center, Zagreb, Croatia

**Keywords:** Primary testicular lymphoma, DLBCL, Cancer/testis antigens, MAGE-A, NY-ESO-1, Immunotherapy

## Abstract

**Background:**

Primary testicular lymphoma (PTL) is a rare and lethal disease. The most common histological subtype is diffuse large B-cell lymphoma (DLBCL). Standard treatments are frequently ineffective. Thus, the development of novel forms of therapy is urgently required. Specific immunotherapy generating immune responses directed against antigen predominantly expressed by cancer cells such as cancer-testis antigens (CTA) may provide a valid alternative treatment for patients bearing PTL, alone or in combination with current therapies.

**Methods:**

Three monoclonal antibodies (mAbs), 77B recognizing MAGE-A1, 57B recognizing an epitope shared by multiple MAGE-A CTA (multi-MAGE-A specific) and D8.38 recognizing NY-ESO-1/LAGE-1 were used for immunohistochemical staining of 27 PTL, including 24 DLBCL.

**Results:**

Expression of MAGE-A1 was infrequently detectable in DLBCL specimens (12.50%), whereas multi-MAGE-A and NY-ESO-1/LAGE-1 specific reagents stained the cytoplasms of tumor cells in DLBCL specimens with higher frequencies (54.17% and 37.50%, respectively) with different expression levels.

**Conclusions:**

These results suggest that MAGE-A and NY-ESO-1/LAGE-1, possibly in combination with other CTA, might be used as targets for specific immunotherapy in DLBCL.

## Background

Primary testicular lymphoma (PTL) is an uncommon and deadly disease accounting for 3-9% of testicular neoplasms [1]. It is mainly a disease of elderly [1], since it is the most common testicular malignancy in men aged over 60 years [2]. Even though bilateral disease also exists, the usual presentation is a painless unilateral enlargement of testis [3]. The pathological diagnosis is mostly obtained after orchiectomy and subsequent histologic examination of the tissue specimen [4]. A majority of patients presents with localized stage I and II PTL[1]. The most common histological subtype of PTL is diffuse large B-cell lymphoma (DLBCL) [5-7]. Other histological subtypes are follicular, plasmocytoma, lymphoblastic and Burkitt’s like lymphoma [2,8,9].

Primary testicular DLBCL is a very aggressive malignancy with a poor outcome, and most patients experience relapse within the first 2 years [10]. Standard treatments, especially for limited disease, are controversial [6]. Results of first-line treatment based on orchiectomy and including a variety of chemotherapy protocols such as R-CHOP (Rituximab – cyclophosphamide hydroxydaunorubicin oncovin prednisone) and/or radiation [10] remain poor[11]. In addition, due to the low incidence of the disease, there is an obvious lack of prospective studies [12]. Consequently, the development of innovative treatment options is required.

Active specific tumour immunotherapy generating immune responses directed against antigens predominantly expressed by cancer cells may provide a valid alternative treatment for patients bearing PTL, alone or in combination with current therapies.

Cancer/testis antigens (CTA) are a category of tumor-associated antigens expressed in a restricted number of healthy tissues such as testicular germ cells, thymus and placenta [13,14], as well as in a large variety of tumors of unrelated histological origin including lymphoma [15]. Because of this expression pattern and of their capability to induce humoral and cellular immune responses, they are considered as relevant targets in cancer immunotherapy [16].

Most probably because of low incidence, the expression of CTA in PTL was not investigated so far. In the present study, we have evaluated by immunohistochemistry (IHC) the expression of different CTA members in PTL. Our results demonstrate that MAGE-A and NY-ESO-1/LAGE-1 expression is detectable in the cytoplasm of tumor cells from DLBCL specimens. These results suggest that these antigens might be potentially used as novel targets for specific immunotherapy.

## Methods

### Tissue samples

We investigated a consecutive series of 24 samples from patients diagnosed for DLBCL at the Department of Urology of the University Hospital of Zagreb (Croatia), of the Clinical Hospital Center of Rijeka (Croatia), of the Clinical Hospitals of Split and Osijek (Croatia) from 1998 to 2008. In addition, 3 samples from patients diagnosed for follicular lymphoma (FL), small lymphocytic lymphoma (SLL) and B-lymphoblastic lymphoma (B-LBL) were also analyzed. Patients underwent conventional orchiectomy and the testis tissue samples were processed for histology as described below.

The study was conducted in accordance with the Declaration of Helsinki and approved by the ethical committee of the University Hospital Center of Zagreb (N°8.1-09/67-2).

### Identification of testicular lymphomas subtypes

IHC was performed using routine diagnostic methods [17]. Briefly, testicular tumor tissues obtained after orchiectomy were formalin-fixed, embedded in paraffin and cut into 4 μm thick sections. Haematoxylin and eosin (HE) staining was used to evaluate morphology. Different lymphoma subtypes were identified by IHC, based on the expression of CD20 (clone L26, 1:50 dilution, Dako, Denmark), CD3 (clone F7.2.38, 1:50 dilution, Dako, Denmark), CD10 (clone 56C6, 1:50, dilution Novocastra, UK), Bcl-6 (clone PG-B6p, 1:10 dilution, Dako, Denmark), CD5 (clone 4C7, 1:50 dilution, Novocastra, UK), Cyclin D1 (clone SP4, 1:25 dilution, Lab Vision/Neomarkers, Thermo Scientific, Fremont, CA), Terminal deoxynucleotidyl transferase (TdT, polyclonal, 1:10 dilution, Dako, Denmark)) and Bcl-2 (clone 124, 1:50 dilution, Dako, Denmark) using avidin-biotin method.

All samples were screened for the presence of tumors by experienced pathologist in the Clinical Hospital Center Zagreb and classified according to the World Health Organization (WHO) criteria.

### Immunohistochemistry

Formalin-fixed paraffin embedded PTL sections were stained with MAGE-A1 specific mAb 77B[18], with multi-MAGE-A specific mAb 57B [19] generated by using recombinant MAGE-A3 as immunogen and recognizing an epitope common to highly homologous MAGE-A3 and MAGE-A4, but also to MAGE-A1, -A2-, A6 and -A12 molecules, and with NY-ESO-1/LAGE-1 specific mAb D8.38 [20]. Monoclonal antibodies were used in the form of undiluted hybridoma supernatants.

Briefly, deparaffinized sections were incubated in citrate buffer (10 mmol/L, pH 6.0), washed with phosphate-buffered saline (PBS) buffer (pH 7.2). Endogenous peroxidase activity was blocked by treatment with Peroxidase-Blocking Solution Dako REAL™ (Dako, Denmark) in accordance to the instructions from the producers. Slides were washed with PBS buffer and incubated for 90 minutes with 77B, 57B, or D8.38 mAbs undiluted supernatants at room temperature. After PBS wash, slides were labeled with streptavidin biotin reagents (Universal Dako LSAB®, Dako, Denmark) and washed. Dako Liquid Dab+ Substrate-chromogen system (Dako, Denmark) was added to the slides before one wash in distilled water. Nuclei were counterstained using Dako REAL™ Hematoxylin (Dako, Denmark). Slides were finally washed again with water, dehydrated with alcohol (96%) and cleared with xylene. Melanomas and testicular tissues expressing CTAs were used as positive controls throughout the study, and healthy skin tissue and unstained tumor cells served as the negative control.

Samples were then classified based on semi-quantitative 0–3 staining score if the expression of CTAs was detectable in <10% of tumor cells (score 1), in 10–50% of tumor cells (score 2), or in >50% of tumor cells (score 3). Score 0 was attributed to negative samples.

## Results

### Histological profiles of the patients

Testis tissue sections from twenty-seven adult male initially referred to urologist for swelling of the testis and undergoing orchiectomy for PTL from 1998 to 2008 were investigated. According to WHO histological classification (Table 1.), of all the patients involved, 24 of 27 had DLBCL, 1 had a follicullar lymphoma (FL), 1 had a small lymphocytic lymphoma (SLL) and 1 had a B-lymphoblastic lymphoma (B-LBL).

**Table 1:** CTA protein expression in PTL subtypes

**Patients**	**Histological subtype**	**MAGE-A1(77B mAb)**			**Multi-MAGE-A(57B mAb)**			**NY-ESO-1(D8.38 mAb)**		
		**% of positive tumor cells**	**Staining score**	**Staining intensity**	**% of positive tumor cells**	**Staining score**	**Staining intensity**	**% of positive tumor cells**	**Staining score**	**Staining intensity**
1	FL	0	0	0	90	3	2	80	3	1
2	SLL	0	0	0	0	0	0	0	0	0
3	B-LBL	0	0	0	100	3	1	0	0	0
4	DLBCL	0	0	0	0	0	0	10	2	1
5	DLBCL	0	0	0	0	0	0	0	0	0
6	DLBCL	0	0	0	0	0	0	0	0	0
7	DLBCL	100	3	2	0	0	0	0	0	0
8	DLBCL	0	0	0	0	0	0	0	0	0
9	DLBCL	0	0	0	0	0	0	0	0	0
10	DLBCL	0	0	0	0	0	0	0	0	0
11	DLBCL	80	3	2	0	0	0	0	0	0
12	DLBCL	0	0	0	0	0	0	100	3	1
13	DLBCL	10	2	1	0	0	0	0	0	0
14	DLBCL	0	0	0	100	3	2	100	3	2
15	DLBCL	0	0	0	100	3	3	100	3	2
16	DLBCL	0	0	0	100	3	2	100	3	2
17	DLBCL	0	0	0	100	3	2	0	0	0
18	DLBCL	0	0	0	100	3	1	0	0	0
19	DLBCL	0	0	0	100	3	2	100	3	3
20	DLBCL	0	0	0	70	3	1	90	3	1
21	DLBCL	0	0	0	100	3	2	100	3	2
22	DLBCL	0	0	0	100	3	3	0	0	0
23	DLBCL	0	0	0	100	3	2	100	3	1
24	DLBCL	0	0	0	90	3	1	0	0	0
25	DLBCL	0	0	0	80	3	2	0	0	0
26	DLBCL	0	0	0	0	0	0	0	0	0
27	DLBCL	0	0	0	100	3	1	0	0	0

DLBCL (Diffuse Large B-cell Lymphoma), FL (Follicular Lymphoma), SLL (Small Lymphocytic Lymphoma), B-LBL (B-Lymphoblastic Lymphoma).

Staining score (percentage of positive tumor cell): 0 (negative), 1 (<10%), 2 (10–50%), 3 (>50%).

Staining intensity: 0 (negative), 1 (weak), 2 (moderate intensity), 3 (strong intensity).

Due to the multi-institutional nature of this study and the long time frame of the study (10 years), age, Ann Arbor tumor stage and local invasion status were not available.

### Immunohistochemical detection of CTA in DLBCL tissues

**Figure 1:**
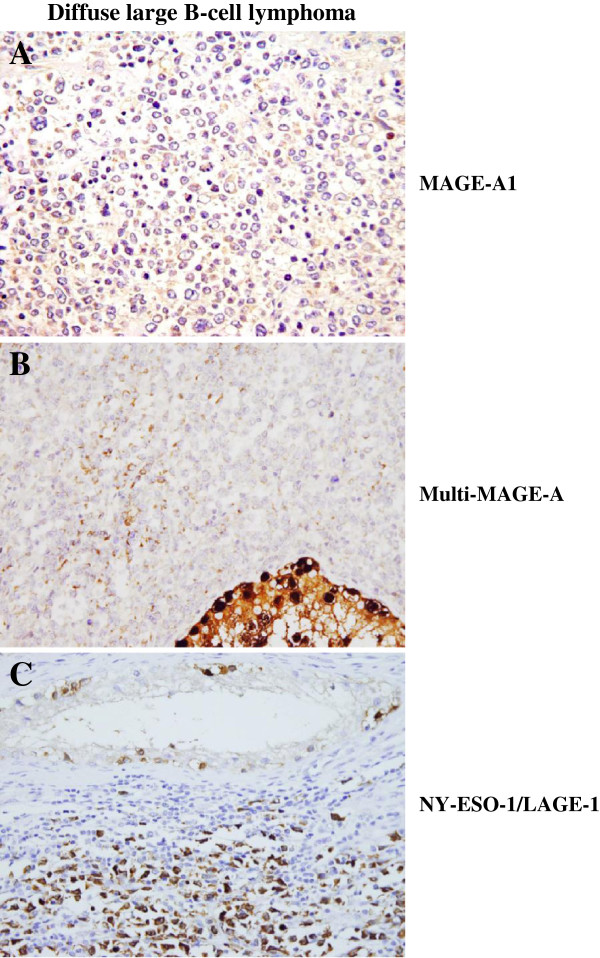
**Immunohistochemical detection of MAGE-A1, multi-MAGE-A, and NY-ESO-1/LAGE-1 in diffuse large B-cell lymphoma tissues.** DLBCL tissues were stained either with MAGE-A1 specific mAb 77B (**A**.), or multi-MAGE-A specific mAb 57B (**B**.) or with NY-ESO-1/LAGE-1 specific mAb D8.38 (**C**.). Representative staining displaying positivity for these CTA are shown.

In order to define if CTA might be relevant targets for specific immunotherapy in DLBCL, we investigated the expression level of MAGE-A1 protein by IHC on paraffin embedded DLBCL tissues sections using MAGE-A1 specific mAb 77B [18]. MAGE-A1 was detectable in 3 of 24 (12.50%) DLBCL specimens (Table 1). As shown in Figure 1A, MAGE-A1 had a clear cytoplasmic location, as already described in melanoma cell line MZ-2 [18]. Staining intensity was weak in 1 and moderate in 2 tumor specimens (Table 1). As shown in Table 1, in one sample, 10–50% of tumours cells were MAGE-A1 positive, whereas in the other two over 50% of tumor cells showed evidence of positive staining.

We concomitantly evaluated multi-MAGE-A specific staining by using 57B mAb [19]. Positive tumor cells were detectable in 13 of 24 (54.17%) DLBCL specimens (Table 1) with a clear cytoplasmic reaction (Figure 1B). Staining intensity was weak in 4, moderate in 7 and strong in 2 specimens (Table 1). Staining score (Table 1), showed that more than 50% of tumor cells (range: 70–100%, Table 1) were MAGE-A positive.

In parallel, we evaluated the expression of NY-ESO-1/LAGE-1, a CTA known to be expressed notably in testicular carcinomas [20], using the previously described NY-ESO-1/LAGE-1 specific mAb D8.38 [20]. As shown in Table 1 and Figure 1C, NY-ESO-1/LAGE-1 protein was detectable in the cytoplasm of tumor cells from 9 of 24 (37.50%) DLBCL samples. Staining intensity was weak in 4, moderate in 4 and strong in 1 tumor specimens (Table 1). As shown in Table 1, except for one DLBCL tissue displaying only 10% of NY-ESO-1/LAGE-1 positive tumor cells, all of DLBCL tissues positive for this antigens showed more than 50% of positive tumor cells (range: 90–100%, Table 1).

As expected, CTA specific staining was occasionally detectable simultaneously in neoplastic cells and in normal spermatogonia (Figure 1.).

Interestingly, the FL was positive for multi-MAGE-A staining and NY-ESO-1/LAGE-1 and negative for MAGE-A1 expression. The B-LBL was positive for multi-MAGE-A but not for MAGE-A1 or NY-ESO-1/LAGE-1 expression, whereas the SLL was negative for all CTA under investigation.

### Co-expression of MAGE-A and NY-ESO-1/LAGE-1 CTA in DLBCL tissues

**Figure 2:**
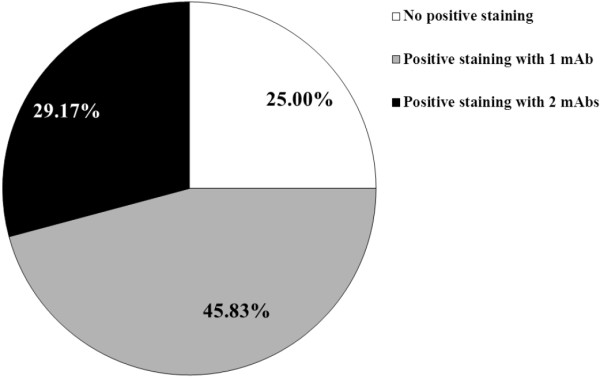
**Percentages of DLBCL samples showing evidence of positive staining upon incubation with CTA specific mAbs.** Testis sections from DLBCL tissues were stained, as described in materials and methods, by IHC with 77B, 57b and D8.38 CTA specific mAbs. DLBCL samples were categorized by taking into account the number of positive reactions per tissue.

Co-expression of NY-ESO-1/LAGE-1 CTA and MAGE-A members might result of particular interest for the design of multi-antigen vaccines. Prompted by this consideration, we evaluated the expression of NY-ESO-1/LAGE-1 as associated with MAGE-A CTA expression in DLBCL tissues. As shown in Figure 2 and Table 1, 18 of 24 (75.00%) DLBCL specimens showed evidence of positive staining following incubation with at least one of the mAbs under investigation and 7 of 24 (29.17%) positivity to at least two reagents could be observed (Figure 2).

## Discussion

Diffuse large B-cell lymphoma (DLBCL) is an infrequent malignancy of the testis [1]. Despite orchiectomy and the administration of aggressive chemotherapy such as R-CHOP, the prognosis is very poor [10]. Therefore, the development of novel treatments is urgently required.

CTA exhibit a restricted normal tissue expression and a widespread expression in tumors of different histological origin. Therefore, CTA may serve as targets for anti-cancer active specific immunotherapy [16]. To date, several CTA families have been identified including MAGE-A and NY-ESO-1/LAGE-1[21]. CTA expression has previously been detected in Reed-Sternberg cells [22]. Furthermore, non-Hodgkin lymphomas [23] and in particular cutaneous lymphomas were also found to express CTA [24]. Most recently, MAGE-A3 gene expression in peripheral blood has been suggested to represent an useful marker for the follow-up of patients with non-Hodgkin lymphoma undergoing chemotherapy [25]. Nevertheless, due to rareness of the disease, nothing is known about CTA expression in DLBCL so far.

In this study, taking advantage of the availability of specific mAbs allowing the identification of CTA-expressing cells in paraffin embedded clinical specimens [18-20], we investigated the expression of MAGE-A and NY-ESO-1/LAGE-1 at the protein level in DLBCL tissues.

Here, we report for the first time CTA expression in diffuse large B-cell lymphoma. In particular, multi-MAGE-A and NY-ESO-1/LAGE-1 specific staining is detectable in the cytoplasm of tumor cells, in more than half and in more than one third of DLBCL samples, respectively. This finding is of particular interest because of the co-existence within the same tissue of healthy cells of the germinal lineage and of neoplastic lymphoma cells. In addition, our results reveal that one third of DLBCL specimens are positive for both MAGE-A and NY-ESO-1 CTA, suggesting that design of multi-antigen vaccines might be of relevance for DLBCL treatment.

## Conclusions

Our work indicates that MAGE-A and NY-ESO-1/LAGE-1 antigens, possibly in combination with other CTA, might represent a realistic therapeutic option in this rare disease.

## Abbreviations

B-LBL: B-lymphoblastic lymphoma; CTA: Cancer/testis antigen; DLBCL: Diffuse large B-cell lymphoma; FL: Follicular lymphoma; IHC: Immunohistochemistry; LAGE-1: L antigen family member-1; mAb: Monoclonal antibody; MAGE-A: Melanoma antigen-A; NY-ESO-1: New York eosophageal squamous cell carcinoma-1; PTL: Primary testicular lymphoma; R-CHOP: Rituximab – cyclophosphamide hydroxydaunorubicin oncovin prednisone; SLL: Small lymphocytic lymphoma; WHO: World Health Organization.

## Competing interests

The authors declare that they have no competing interests.

## Authors’ contributions

TH conceived the study, and acquired samples and data. ZK participated to the coordination of the study and participated to immunohistological staining and data interpretation. II carried out the immunohistochemical staining, the morphological analysis, and the scoring of the sections. KL-H critically revised the manuscript. MR made substantial contributions to the interpretation of data. NB-J participated in the interpretation of data. GCS generated the antibodies, contributed to conception and design of the study and revised the manuscript. AJ participated in the design of the study. CM drafted the manuscript and figures, participated to the design of the study and analysed the data. All authors read and approved the final manuscript.
